# A Gut Symbiotic Bacterium *Serratia marcescens* Renders Mosquito Resistance to *Plasmodium* Infection Through Activation of Mosquito Immune Responses

**DOI:** 10.3389/fmicb.2019.01580

**Published:** 2019-07-18

**Authors:** Liang Bai, Lili Wang, Joel Vega-Rodríguez, Guandong Wang, Sibao Wang

**Affiliations:** ^1^School of Life Science and Technology, Tongji University, Shanghai, China; ^2^CAS Key Laboratory of Insect Developmental and Evolutionary Biology, CAS Center for Excellence in Molecular Plant Sciences, Institute of Plant Physiology and Ecology, Shanghai Institutes for Biological Sciences, Chinese Academy of Sciences, Shanghai, China; ^3^University of Chinese Academy of Sciences, Beijing, China; ^4^Laboratory of Malaria and Vector Research, National Institute of Allergy and Infectious Diseases, National Institutes of Health, Rockville, MD, United States

**Keywords:** *Anopheles* mosquito, gut symbiont, antiparasitic defense, malaria transmission, immune activation

## Abstract

The malaria development in the mosquito midgut is a complex process that results in considerable parasite losses. The mosquito gut microbiota influences the outcome of pathogen infection in mosquitoes, but the underlying mechanisms through which gut symbiotic bacteria affect vector competence remain elusive. Here, we identified two *Serratia* strains (Y1 and J1) isolated from field-caught female *Anopheles sinensis* from China and assessed their effect on *Plasmodium* development in *An. stephensi*. Colonization of *An. stephensi* midgut by *Serratia* Y1 significantly renders the mosquito resistant to *Plasmodium berghei* infection, while *Serratia* J1 has no impact on parasite development. Parasite inhibition by *Serratia* Y1 is induced by the activation of the mosquito immune system. Genome-wide transcriptomic analysis by RNA-seq shows a similar pattern of midgut gene expression in response to *Serratia* Y1 and J1 in sugar-fed mosquitoes. However, 24 h after blood ingestion*, Serratia* Y1 modulates more midgut genes than *Serratia* J1 including the c-type lectins (CTLs), CLIP serine proteases and other immune effectors. Furthermore, silencing of several *Serratia* Y1-induced anti-*Plasmodium* factors like the thioester-containing protein 1 (TEP1), fibrinogen immunolectin 9 (FBN9) or leucine-rich repeat protein LRRD7 can rescue parasite oocyst development in the presence of *Serratia* Y1, suggesting that these factors modulate the *Serratia* Y1-mediated anti-*Plasmodium* effect. This study enhances our understanding of how gut bacteria influence mosquito-*Plasmodium* interactions.

## Introduction

Malaria continues to be one of the most devastating infectious diseases and a major public health problem in tropical and subtropical regions. The increase in mosquito insecticide resistance and parasite drug resistance, combined with the lack of an effective vaccine, have stalled the steady reduction in global malaria cases (World Health Organization, 2018), making the development of new malaria interventions a global priority.

Malaria is caused by *Plasmodium* parasites and is transmitted to humans by *Anopheles* mosquitoes. The most severe bottleneck during *Plasmodium* development occurs in the mosquito midgut, where the majority of ingested parasites are killed (Vaughan et al., 1992; Ghosh et al., 2000; Pradel, 2007; Simon et al., 2009). The malaria parasite encounters a hostile environment in the mosquito midgut and undergoes a chain of complex developmental transitions that are required for successful transmission (Smith et al., 2014). Several reports have shown that the mosquito midgut microbiota affects mosquito susceptibility to parasite infection (Pumpuni et al., 1993, 1996; Dong et al., 2009; Cirimotich et al., 2011; Boissiere et al., 2012; Gendrin and Christophides, 2013; Bahia et al., 2014; Smith et al., 2014; Stathopoulos et al., 2014). Diverse species of bacteria colonize the midgut of both laboratory-reared and field caught mosquitoes, and some can inhibit *Plasmodium* development (Pumpuni et al., 1996; Straif et al., 1998; Gonzalez-Ceron et al., 2003). Likewise, elimination of the gut bacteria with antibiotics renders the mosquito more susceptible to *Plasmodium* infection, which can be reverted by reintroduction of bacteria in the midgut (Dong et al., 2009; Gendrin et al., 2015). However, the mechanisms by which the specific gut bacteria negatively impact malaria parasite development in the mosquito midgut are not completely understood.

The number of bacteria in the mosquito midgut increases exponentially within 24 h of a blood meal ingestion (Pumpuni et al., 1996; Wang et al., 2012), resulting in induction of the midgut immune responses (Dong et al., 2009; Meister et al., 2009). This immune activation can also modulate the mosquito defense against the malaria parasite (Meister et al., 2005, 2009; Bahia et al., 2014). RNA transcription profiling of microbe-free aseptic and septic mosquitoes identified many genes up-regulated by gut bacteria, including several anti-*Plasmodium* factors (Dong et al., 2009). Although, there is overlap between the mosquito antibacterial and anti-malarial immune responses, some antibacterial immune genes have no impact on *Plasmodium* development (Dimopoulos et al., 1997; Oduol et al., 2000; Dong et al., 2006, 2009). In addition, the effect of the gut microbiota on *Plasmodium* infection may be exerted through direct interactions with bacteria-produced anti-*Plasmodium* factors or by the formation of a physical barrier that blocks the parasite’s access to the midgut epithelium (Cirimotich et al., 2011; Bando et al., 2013; Bahia et al., 2014; Song et al., 2018). Conversely, a positive correlation was reported between the presence of *Enterobacteriaceae* bacteria in the midgut of field-caught mosquitoes and the *Plasmodium falciparum* infection status (Boissiere et al., 2013). These indicate that the effect of mosquito gut bacteria on parasite infection is complex and may depend on species-specific or strain-specific interactions.

In this study, we examined the influence of two *Serratia* strains isolated from field-caught *Anopheles sinensis*, the main malaria vector in Asia, on *Plasmodium* infection. *Serratia* strain Y1 inhibited *Plasmodium berghei* infection in *An. stephensi* mosquitoes, whereas *Serratia* strain J1 had no impact on parasite infection. Gene expression and RNA interference analysis show that the inhibition of *Plasmodium* by *Serratia* Y1 is mediated by the bacterial activation of the mosquito immune system. Understanding the interactions and mechanisms through which gut commensal bacteria actively shape mosquito immunity may yield new insights into vector-pathogen interactions and may help in the development of new vector-based disease interventions.

## Materials and Methods

### Ethics Statement

This study was carried out in strict accordance with the guidelines of the Shanghai Institutes for Biological Sciences Animal Care and Use Committee, and all animal work was approved by the committee.

### Mosquito Rearing, Oral Bacterial Introduction and Infection with *Plasmodium berghei*

*Anopheles stephensi* (Nijmegen strain) and *An. sinensis* (Jiangsu strain) mosquitoes were maintained at 27°C with 70 ± 5% relative humidity under 12 h/12 h day-night cycle. Adult mosquitoes were maintained on 10% (w/v) sucrose. The larvae were reared on cat food pellets and ground fish food supplement. Axenic female mosquitoes were generated *via* treatment with oral antibiotics as previously described (Wei et al., 2017). Female mosquitoes were reared with 10% sucrose solution containing penicillin (10 unit/ml), streptomycin (10 μg/ml) and gentamicin (15 μg/ml). Three days after antibiotic treatment, the antibiotic solution was replaced by sterile water, and mosquitoes were starved for 8 h and fed for 24–48 h on a cotton pad soaked with a 5% sucrose solution containing *Serratia* bacteria at a final concentration of 10^7^ CFU/ml. The control group was only given 5% sucrose solution without any bacteria. The mosquitoes were allowed to feed on wild-type *P. berghei*-mCherry (Graewe et al., 2009) infected mouse. On each experiment, control and experimental groups were fed on the same infected mouse. Fully engorged mosquitoes were separated within 24 h and provided with a cotton pad soaked with 5% (w/v) sterile sucrose solution that was replaced twice a day. Oocyst numbers in midguts were determined on day 10 after infection, stained with 0.1% (w/v) mercurochrome.

### Direct PCR Amplification of the 16S rRNA Gene was Performed from the Isolated Bacteria

#### 16S rRNA Gene Cloning and Sequencing

A fragment of approximately 1.5 kb of the 16S rRNA gene was PCR amplified from isolated bacteria from the mosquito midgut using the following primers: 27F (5′-AGAGTTTGATCMTGGCTCAG-3′) and 1492R (5′-GGTTACCTTGTTACGACTT-3′). PCR mixtures used to amplify bacterial sequences contained about 50 ng DNA; 0.4 mM each dNTP; 2.5 U Taq DNA polymerase; 3 mM MgCl2 (50 mM magnesium chloride); 1X PCR buffer (200 mM Tris, pH 8.4, 500 mM KCl); 25 pmol of each primer and sterile water to a final volume of 50 μl. PCR amplification was carried out with the following conditions: 95°C for 3 min; 35 cycles at 95°C for 15 s, 58°C for 30 s and 72°C for 2 min, and a final extension at 72°C for 5 min. Reactions were carried out in an ProFlex PCR System (Applied Biosystems). Amplification products were analyzed by electrophoresis on a 1% agarose gel and visualized under UV light after staining with ethidium bromide. PCR products were purified using the Wizard® SV Gel and PCR Clean-Up System (Promega, Madison, WI, USA) according to the manufacturer recommendations. Purified PCR products were cloned into a pGEM-T easy vector according to manufacturer instructions and transformed into competent DH5α *Escherichia coli* cells by heat-shock for 90 s. Recombinant colonies were identified using blue and white screening on LB agar medium containing 100 μg/ml ampicillin, 2 mM IPTG, and 0.004% X-gal. The plates were incubated at 37°C overnight. The white colonies were selected and cultured in Luria-Bertani (LB) broth complemented with 100 μg/ml ampicillin. Plasmid extraction were done using an alkaline lysis method. The 16S rRNA gene was sequenced by Sangon Biotech (Shanghai, China) Co., Ltd. The sequences obtained were compared with GenBank database for bacterial species identification.

#### Generation of Fluorescent Strains of *Serratia* Y1 and J1

To integrate an enhanced fluorescent protein gene (*egfp*) into the chromosome of *Serratia* Y1 and J1, the transposon plasmid pBAM2-GFP (Wang et al., 2017) was transformed into a donor strain *E. coli* S17-1λpir. Freshly cultured transformed donor strain and the recipient strains (*Serratia* Y1 and J1) cells were washed and resuspended with 10 mM MgSO4 solution to a final OD600 of 0.1 of each strain, mixed (1:1) and co-cultured on Luria-Bertani (LB) agar plates at 37°C for conjugation mating. After 5 h incubation, the fluorescence gene was mobilized and integrated into the genome of the recipient strain *Serratia* Y1 and J1. The co-culture was seriously diluted and plated on LB agar plates containing 100 μg/ml of kanamycin. The plates were incubated overnight at 30°C and fluorescent colonies were identified under fluorescent microscopy.

#### Colonization of *Serratia* Y1 and J1 in the Mosquito Midgut

To test colonization of *Serratia* Y1 and J1 in adult *An. sinensis* and *An. Stephensi* mosquitoes, GFP-labeled *Serratia* strains (Y1-GFP and J1-GFP) were used in this assay. The bacteria were cultured overnight in LB broth medium at 30°C, washed twice in sterile 1 × PBS and resuspended in 5% sterile sucrose solution to obtain 1 × 10^7^ cells/ml. The bacteria were introduced into 2 day-old female mosquitoes by feeding on a cotton pad moistened with bacterial suspension for 24–48 h. Two days later, the mosquitoes were allowed to feed on a non-infected mouse. Before and post-blood meal, 10 mosquitoes were collected at different time points, and were surface-sterilized by washing in 75% ethanol for 3 min and then rinsing them in sterile PBS three times. The midguts were dissected under sterile conditions and homogenized in 100 μl sterile PBS. The bacterial load was determined by plating 10-fold serial dilutions of the homogenates on LB agar plates containing 100 μg/ml of kanamycin and incubating the plates overnight at 30°C. The fluorescent colonies were counted by fluorescent microscopy.

#### RNA Extraction and Transcriptome Sequencing

*Serratia* Y1 and J1 were separately fed to 3-day-old aseptic mosquitoes in a sugar meal for 24–48 h, then mosquitoes were allowed to feed on non-infected blood. About 50 female mosquitoes were collected from two biological replicates before (0 h) and 24 h after blood meal. The aseptic females that did not feed on Y1 and J1bacteria were used as the controls. Mosquito midguts were dissected in ice-cold PBS and total RNA was extracted using Direct-zol RNA Miniprep Kit (The Epigenetics Company, USA) followed by RNase-free DNase I treatment. Messenger RNA (mRNA) was purified, and reverse-transcribed into cDNA libraries using the NEBNext^®^ Ultra™ RNA Library Prep Kit for Illumina^®^ (NEB, Boston, Massachusetts, USA). The cDNA libraries were sequenced on an Illumina HiSeq 2000 platform.

### Assembly and Annotation of Transcriptomes

Before assembling the clean reads, the raw reads were preprocessed using filter-fq software. For the transcriptome analysis, the clean reads were aligned in paired-end mode against the *Anopheles stephensi* SDA-500 genome^1^ using HISAT v0.1.6-beta and assembled with Cufflinks with the default settings^2^. The gene annotation was performed by FunCat^3^. Secreted proteins were predicted by SignalP 3.0^4^.

#### Differential Expression Genes, Clustering and Functional Enrichment Analysis

Differential expression genes (DEGs) were identified for each time point, which were compared to the data of the control group using Cuffdiff. Differential expression was detected using log2 (fold change) ≥ 1.0 and adjusted *p* < 0.05. The combined transcriptomes were used as the background to search for GO terms enriched within the DEGs using http://bioinfo.cau.edu.cn/agriGO/ and a *p* < 0.01 as the parameters for determining significantly enriched terms. Similarly, pathways significantly enriched with the DEGs were identified by mapping all DEGs to terms in the KEGG database using KOBAS2.0 with a *p* < 0.05.

#### Validation of DEGs by qRT-PCR Analysis

First-strand cDNA was synthesized from total RNA using the PrimeScript RT Reagent Kit with gDNA Eraser (Takara) according to the manufacturer’s instructions. Quantitative real-time PCR (qRT-PCR) analysis was performed with the PikoReal 96 (Thermo, USA) using the AceQ qPCR SYBR Green Master Mix (Vazyme). The housekeeping As S7 gene was used as an endogenous control. The primers are shown in Supplementary Table S2.

#### Double-Stranded RNA Synthesis and Gene Silencing in Adult Mosquitoes

Forward and reverse primers with T7 promoter sequence (5′-TAATACGACTCACTATAGGG-3′) were used to amplify the fragment of the enhanced green fluorescent protein (eGFP) gene from plasmid pBacRMCE-Ac5-EGFP (Supplementary Table S3). Fragments of the *Rel1*, *Rel2*, *TEP1*, *FBN9*, and *LRRD7* genes were amplified from *An. stephensi* cDNA. Purified PCR products were used as template for *in vitro* double-strand RNA (dsRNA) transcription using the MEGAscript RNAi Kit (Thermo Fisher Scientific). Pure dsRNAs were diluted to approximately 3 μg/μl in DEPC-water. Two-day-old female mosquitoes were intrathoracically injected with 138 nl of either dsRel1, dsRel2, dsTEP1, dsFBN9, dsLRRD7, or dsGFP as a control. The silencing efficiency was evaluated by qPCR at selected time points after injection.

### Statistical Analysis

Significant difference in oocyst intensity between two samples was analyzed using the Mann-Whitney test. The statistics were performed using GraphPad Prism version 5.00 for Windows (GraphPad Software). *p* < 0.05 was considered to be statistically significant.

## Results

### Effect of *Serratia* Strains on *Plasmodium Berghei* Infection in the Mosquito

**Figure 1:**
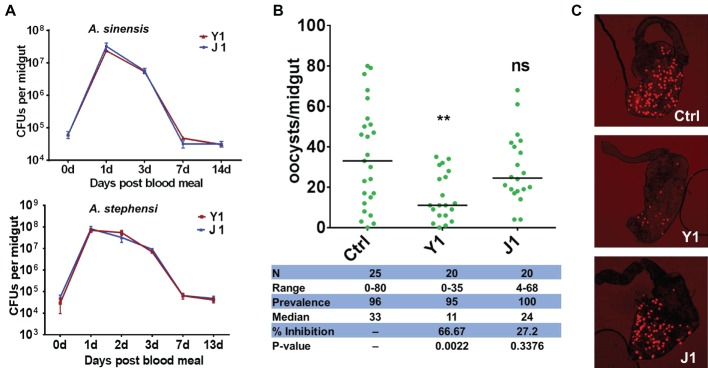
Effect of different *Serratia* strains on *Plasmodium berghei* oocyst development. **(A)** *Serratia* strains Y1 and J1 stably colonize the midgut of female *An. sinensis* and *An. stephensi* mosquitoes and rapidly proliferate after a blood meal. The eGFP-tagged strains Y1-GFP and J1-GFP were fed to 3-day-old female *An. sinensis* and *An. stephensi* mosquitoes in a 5% sugar meal for 24 h, then mosquitoes were allowed to feed on a blood meal. Fluorescent bacteria colony-forming units (CFUs) were determined by plating serially diluted homogenates of midguts on LB agar plates containing 100 mg/ml of kanamycin. Data were pooled from three biological replicates (shown are means ± SEM). **(B)** Oocyst infection intensity in *An. stephensi* mosquitoes colonized with *Serratia* Y1 or J1 after feeding on *P. berghei* mCherry infected mice. Each dot represents the oocysts number from individual midguts, and the horizontal lines indicate the median number of oocysts. Inhibition = [(# of oocyst in control group – # of oocyst in experimental group)/# of mosquitoes in control group] × 100; Median, median oocyst number per midgut; N, number of mosquitoes analyzed; Prevalence, percentage of mosquitoes carrying at least one oocyst; Range, range of oocyst numbers per midgut. The experiments were repeated three times with similar results (Supplementary Figure S2). The Mann-Whitney test was used to determine significance in oocysts numbers, **p < 0.01. **(C)** mCherry oocysts in the midgut epithelium of mosquitoes that had been fed with only sugar solution (top panel), *Serratia* Y1 (center panel) or J1 (bottom panel).

We isolated two dominant *Serratia* strains, termed Y1 and J1, from the midguts of field-collected adult female *An. sinensis* mosquitoes in China. The 16S rRNA gene sequence showed 99% similarity to *Serratia marcescens*. The two *Serratia* strains, Y1 and J1, could stably colonize the midgut of both *An. sinensis* and *An. stephensi* mosquitoes, and rapidly proliferate by more than 200-fold 24 h after a blood meal (Figure 1A). These two strains did not significantly affect mosquito survival whether the mosquitoes fed on sugar or blood (Supplementary Figure S1).

The downstream analyses were performed in *An. stephensi* because it is difficult to maintain *An. sinensis* under laboratory conditions. Investigation of the effect of the two *Serratia* strains on *Plasmodium* development in the midgut of *An. stephensi* show that *Serratia* Y1 significantly reduced oocyst formation (*p* < 0.001), while *Serratia* J1 did not affect *P. berghei* oocyst formation (Figures 1B,C; Supplementary Figure S2). Thus, we identified two mosquito *S. marcescens* strains, one of them inhibiting *Plasmodium* infection in the mosquito midgut.

### *Serratia* Y1 Inhibits *Plasmodium* Development Through Activation of the Mosquito Immune System

**Figure 2:**
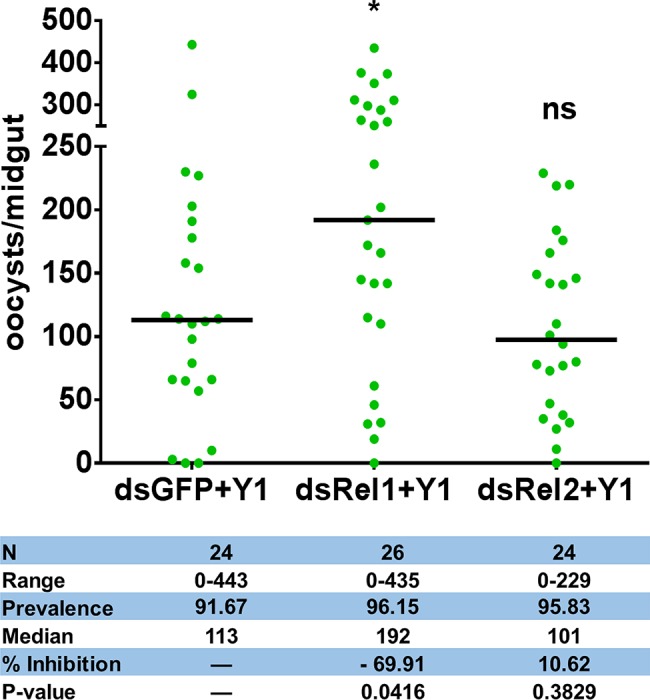
Effect of *Rel1* and *Rel2* silencing on the *Serratia* Y1-mediated anti-*Plasmodium* activity. *Rel1* and *Rel2* were silenced in *An. stephensi* mosquitoes by systemic injection of dsGFP, dsRel1 or dsRel2 RNA. The injected mosquitoes were fed on a sugar meal containing *Serratia* Y1. Three days later, all mosquito groups were allowed to feed on the same *P. berghei* infected mouse. The injected double-stranded RNA (ds) and presence (Y1) of *Serratia* Y1 are indicated below each column. Each dot represents the number of oocysts from an individual midgut, and the horizontal lines indicate the median number of oocysts. The experiments were repeated three times with similar results (Supplementary Figure S3). The Mann-Whitney test was used to determine significance in oocysts numbers, *p < 0.05.

To test whether *Serratia* Y1 exerts the parasite inhibitory effect through stimulation of the mosquito immune system, we used RNA interference (RNAi) to silence the expression of Rel1 and Rel2, the NF-κB transcription factors of Toll and IMD signaling pathways that regulate mosquito innate immunity, respectively. We compared the oocyst numbers in the midguts of *An. stephensi* mosquito cohorts that had been injected with double-stranded RNA either for GFP (dsGFP), dsRel1 or dsRel2. Inhibition of the Toll pathway by Rel1 knockdown significantly increased *P. berghei* infection in mosquitoes fed with Y1 (Figure 2; Supplementary Figure S3). Silencing of Rel2 did not rescue *P. berghei* oocyst development in the presence of Y1 (Figure 2; Supplementary Figure S3). These results suggested that *Serratia* Y1 exerts its anti-*Plasmodium* activity through the activation of the Toll immune pathway.

### Mosquito Global Transcriptome Responses to *Serratia* Challenge

**Figure 3:**
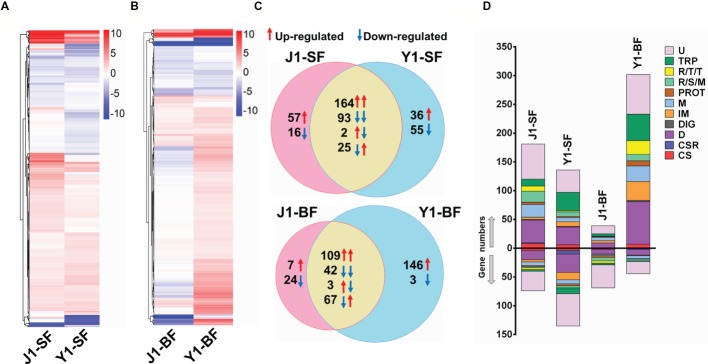
Comparative analysis of transcriptional profiles in mosquito midguts challenged with *Serratia* Y1 or J1. **(A,B)** Heat maps and hierarchical clustering analysis of differentially expressed genes (DEGs) in the midgut of *An. stephensi* mosquitoes fed with *Serratia* Y1 or J1 before (SF, sugar fed) and 24 h after blood meal (BF, blood fed). DEGs in each group were identified with log 2 (fold change) of ≥1 (*p* < 0.05) by comparison of transcript abundance between bacteria challenged and aseptic mosquitoes. Colors from white to red indicate up-regulation; colors from white to blue indicate down-regulation. **(C)** Venn diagram showing the number of shared and specific DEGs in the midgut of mosquitoes fed with *Serratia* Y1 or J1 before and 24 h after blood meal. Red and blue arrows indicate the mosquitoes challenged with *Serratia* Y1 and J1, respectively. **(D)** Global gene regulation in the different groups. Proportions and numbers of genes belonging to distinct functional groups were up- (red arrow) or down-regulated (blue arrow) in the corresponding groups. U, unknown functions; TRP, transport; R/T/T, replication, transcription, translation; R/S/M, oxidoreductive, stress-related and mitochondrial; PROT, proteolysis; M, metabolism; IM, immunity; DIG, digestion; D, diverse; CSR, chemosensory protein; CS, cytoskeletal, structural.

To gain a better understanding of the differences in the mosquito immune response to *Serratia* Y1 and J1, we compared the midgut gene transcriptional profile of mosquitoes colonized by *Serratia* Y1 or J1 before (0 h) and 24 h after a blood meal. RNA-seq data from six RNA libraries are summarized in Supplementary Table S1. Genome-wide transcriptomic profiles showed a similar pattern of midgut gene expression in response to *Serratia* Y1 and J1 in sugar-fed mosquitoes (Figure 3A). A total of 375 genes were differentially regulated in the *Serratia* Y1 challenged group (*p* < 0.05), of which 225 genes were up-regulated and 150 genes were down-regulated (Figure 3C). In the *Serratia* J1 challenged group, 357 genes were differentially regulated including 223 up-regulated and 134 down-regulated genes (Figures 3A,C). The majority of the DEGs (257 genes) had similar expression pattern between the two groups (Figure 3C). At 24 h post-blood meal, *Serratia* Y1 modulated 370 DGEs while *Serratia* J1 regulated only 252 DGEs (Figures 3B,C). Interestingly, a total of 146 upregulated and 3 downregulated genes were specific for the *Serratia* challenged Y1 group. However, the *Serratia* J1 challenged group only showed 7 upregulated and 24 downregulated genes specific to this group (Figures 3B,C). This indicates that *Serratia* Y1 regulates more genes in the midgut after the mosquito takes a blood meal than *Serratia* J1.

Gene Ontology (GO) enrichment analysis was performed to show the potential biological function of the differentially expressed genes. For 0 h before blood meal, we found that, 7 of 48 gene functional groups (14.6%) belonging to extracellular matrix, extracellular region part, extracellular space and transcription regulator were only enriched in the *Serratia* J1 challenged group, while 37 gene functional groups (77%) were enriched in both Y1 and J1 groups (Supplementary Figure S4). At 24 h post-blood meal, 19 of 50 GO terms (38%) were only presented in the *Serratia* Y1 challenged group (Supplementary Figure S5). KEGG classification was also used to analyze the corresponding metabolic pathways involved in the regulation of mosquito genes induced by *Serratia* challenge. Before blood meal, 39 and 42 pathways of level 2 were enriched in the *Serratia* Y1 challenged group and the *Serratia* J1 challenged group, respectively (Supplementary Figure S6A). At 24 h post-blood meal, 42 and 34 pathways of level 2 were enriched in the *Serratia* Y1 challenged group and the *Serratia* J1 challenged group, respectively (Supplementary Figure S6B). Pathways including global and overview maps, signal transduction, immune system, transport and catabolism, signaling molecules and interaction, amino acid metabolism, digestive system and endocrine system were overrepresented in the *Serratia* Y1 challenged group (Supplementary Figure S6B).

### *Serratia* Y1 Modulates Mosquito Immune Genes

**Figure 4:**
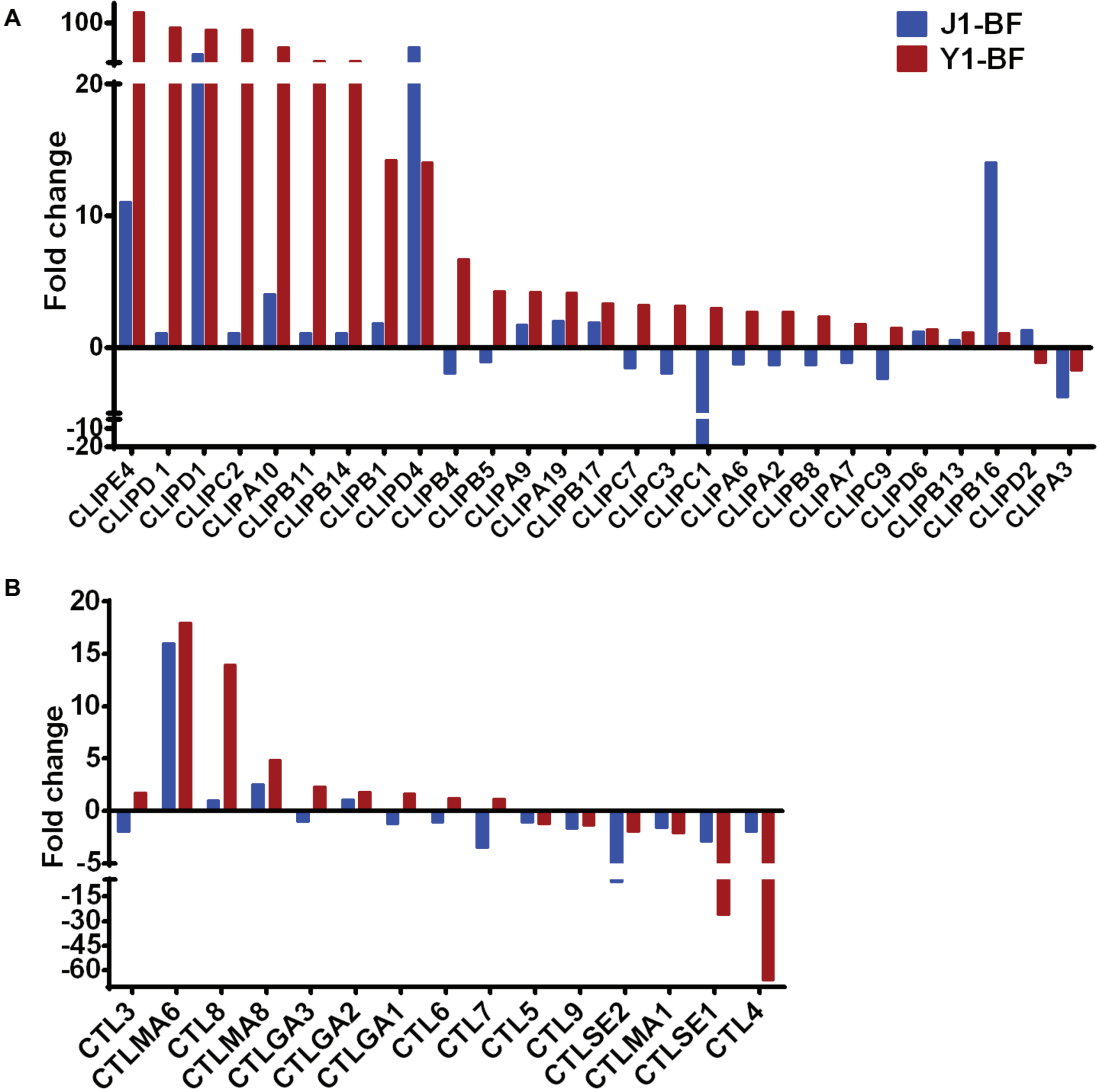
Transcript abundance of CLIP and CTL family genes. **(A)** Fold change in transcript levels of the CLIP family genes in the midgut of mosquitoes fed with *Serratia* Y1 or J1, as compared with the aseptic control mosquitoes. **(B)** Fold change in transcript levels of the CTL family in the midgut of mosquitoes fed with *Serratia* Y1 or J1, relative to the aseptic mosquitoes.

Given that *Serratia* Y1 exerts anti-*Plasmodium* activity by stimulating the mosquito immune pathway (Figure 2), the expression profiles of genes involved in mosquito immunity were further analyzed. In sugar-fed mosquitoes, the number of immune regulated genes was similar between *Serratia* Y1 and *Serratia* J1 colonized mosquitoes (Figure 3D). At 24 h post-blood meal, there were marked differences in immune gene regulation between *Serratia* Y1 and J1 challenged groups. A total of 33 immunity genes were up-regulated in mosquitoes fed with *Serratia* Y1. However, in mosquitoes fed with *Serratia* J1 only 4 immune genes were up-regulated and 1 down-regulated (Figure 3D). In mosquitoes challenged by *Serratia* Y1, the antimicrobial immune genes with the highest upregulation were *Defensin 1*(*DEF1*), *Cecropins 1* (*CEC1*), *Cecropins 2* (*CEC2*) and *Gambicin 1* (*GAM1*). In addition, except for CLIPD 2 and CLIPA 3, all other genes encoding the CLIP family were up-regulated by *Serratia* Y1 (Figure 4A). The expression profiles of CTL family genes were also differently regulated in the midgut of mosquitoes fed with *Serratia* Y1 and J1 (Figure 4B).

### *Serratia* Y1 Modulates the Anti-*Plasmodium* Effector Genes

**Figure 5:**
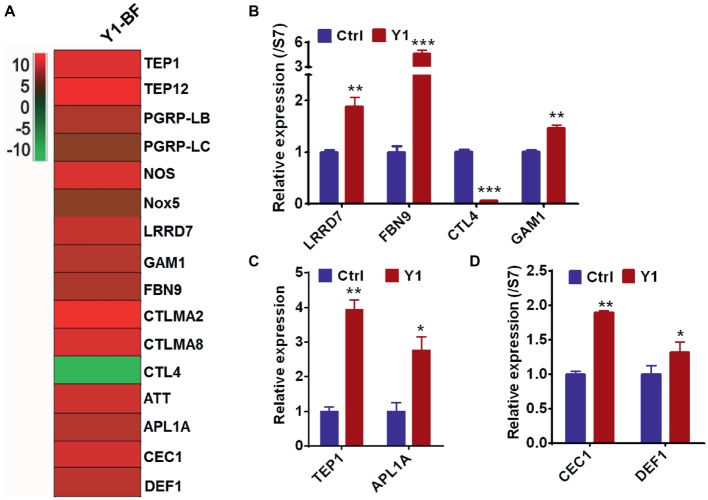
*Serratia* Y1 elicits expression of immune genes in the midgut of mosquitoes 24 h post-blood meal. **(A)** Fold change in transcript levels of immune marker genes in the midgut of Y1-containing mosquitoes 24 h post-blood meal obtained from RNA-seq data. **(B)** Relative expression of immune factor genes (*LRRD7, FBN9, CTL4,* and *GAM1*) in the midgut of mosquitoes 24 h post-blood meal detected by qPCR. **(C)** Relative expression of *TEP1* and *APL1A* in the midgut of mosquitoes 24 h post-blood meal detected by qPCR. **(D)** Relative expression of *DEF1* and *CEC1* in the midgut of mosquitoes 24 h post-blood meal detected by qPCR. mRNA levels of the tested genes were normalized to that of the housekeeping gene S7. Error bars indicate SD of three technical replicates. Experiments were repeated three times with similar results, *p < 0.05; **p < 0.01; ***p < 0.001.

To validate the RNA-seq results, we focused on the transcriptional regulation of several potent anti-*Plasmodium* and anti-bacterial effector genes encoding the thioester-containing protein 1 (TEP1), *Anopheles Plasmodium* responsive leucine-rich repeat protein (APL1A), leucine-rich repeat protein LRRD7, fibrinogen immunolectin 9 (FBN9), *Plasmodium* protective c-type lectin 4 (CTL4) and Gambicin (GAM1). All these genes were differentially regulated in the midgut of mosquitoes fed with *Serratia* Y1 at 24 h post-blood meal (Figure 5A). Real-time quantitative PCR (qPCR) confirmed that *TEP1*, *APL1A*, *LRRD7*, *FBN9*, and *GAM1* were significantly up-regulated, while the protective agonist *CTL4* gene was significantly down-regulated in the mosquitoes fed with Y1 at 24 h post-blood meal (Figures 5A–C). The other two antimicrobial peptides (*DEF1* and *CEC1*) were also significantly up-regulated (Figure 5D).

**Figure 6:**
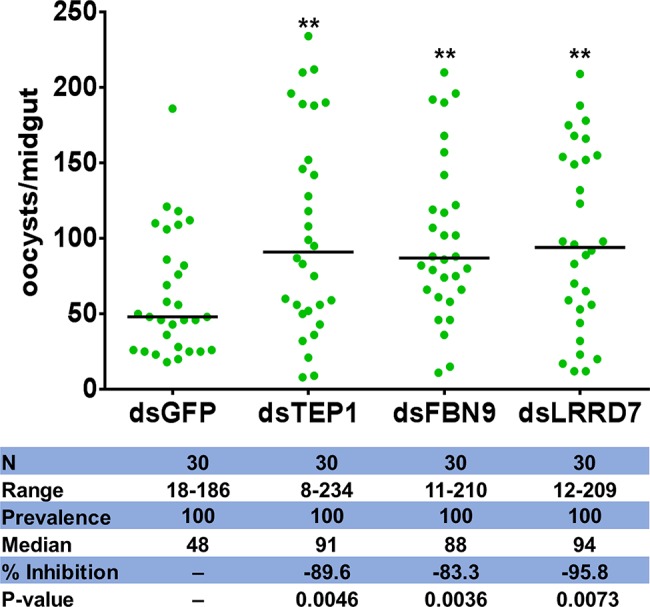
Oocyst loads following silencing of *TEP1*, *FBN9* and *LRRD7* genes. *An. stephensi* female mosquitoes were injected with dsRNA solution for *TEP1*, *FBN9*, *LRRD7* or *GFP* as a control. Injected mosquitoes were colonized with *Serratia* Y1 and then fed on a *P. berghei* infected mouse 3 days after dsRNA injection. Mosquito midguts were dissected 10 days post-blood feeding to determine the number of oocysts per mosquito. Each dot represents the number of oocysts from individual midguts, and the horizontal lines indicate the median number of oocysts. The experiments were repeated three times with similar results (Supplementary Figure S8). The Mann-Whitney test was used to determine significance in oocysts numbers, **p < 0.01.

To establish further evidence that *Serratia* Y1 impacts *P. berghei* infection through activation of anti-*Plasmodium* effector genes, we chose three induced potent anti-*Plasmodium* factors, TEP1, FBN9, and LRRD7 for validation in mosquitoes colonized with *Serratia* Y1. Midgut mRNA levels for *TEP1*, *FBN9*, and *LRRD7* in the dsRNAs-injected mosquitoes were markedly reduced compared to the dsGFP treated control (Supplementary Figure S7). Silencing of the three effector genes resulted in a significant increase of oocyst numbers when compared to the dsGFP -injected control in the *Serratia* Y1 colonized mosquitoes (Figure 6; Supplementary Figure S8). This result indicates that the depletion of dsTEP1, dsFBN9, or dsLRRD7 significantly reverses the refractoriness conferred by *Serratia* Y1 infection.

## Discussion

Mosquito gut bacteria form a resident community that has co-evolved with the insect host. In addition to playing important roles in digestion and harvesting energy, commensal bacteria are important factors determining the outcome of pathogen infection. Other mechanisms such as induction of mosquito innate immune responses have also been proposed, although the exact effect and mode of action by individual commensal bacteria are largely unknown. Interestingly, the presence, outgrowth, or loss of certain bacterial components of gut microbiota correlates with increased or decreased susceptibility to *Plasmodium* and dengue virus infection (Apte-Deshpande et al., 2012, 2014; Boissiere et al., 2012; Bahia et al., 2014). A previous study also showed that different strains of the same bacterial species can induce different outcomes on *Plasmodium* infections (Bando et al., 2013), indicating that the effect of gut bacteria on *Plasmodium* infection is complex and may involve species-specific or strain-specific interactions. In this study, we identified two mosquito symbiotic *Serratia* strains, from field caught *An. sinensis* mosquitoes, with different effect on *Plasmodium* development. *Serratia* Y1 shows anti-*Plasmodium* activity, but *Serratia* J1 does not influence parasite development in the midgut of mosquitoes. In mosquitoes, anti-*Plasmodium* and antibacterial immune defenses are largely controlled by the Toll and Imd immune signaling pathways, and the Toll pathway seems to be most efficient against the rodent parasite *P. berghei* (Meister et al., 2005, 2009; Garver et al., 2009). Some immune factors and AMP genes are regulated by both Toll and Imd pathways (Luna et al., 2006). This dual activation may be modulated by independent stimulation or by cross-regulation of the two signaling pathways (De Gregorio et al., 2002). Previous study also showed that the Toll and Imd pathways can interact synergistically and activate innate immune responses in *Drosophila*, demonstrating that cross-regulation of the two pathways occur (Tanji et al., 2007). Our findings also show that *Serratia* Y1 and J1 induce some genes that are regulated by both Toll and Imd pathways. We further showed that *Serratia* Y1 indirectly antagonizes *P. berghei* infection through activation of the mosquito Toll immune pathway.

Since the midgut is the primary site of response to the invading *Plasmodium*, identification of factors regulated by gut bacteria would provide insight into the mechanisms of how commensal bacteria activate immune-mediated anti-*Plasmodium* activity. Toward this we investigated the influence of *Serratia* Y1- or J1 on the mosquito midgut transcriptome. Before blood meal, the number of regulated immune genes in the *Serratia* Y1 and J1 groups was very similar, which may indicate that the mosquitoes modulate similar basal immune responses to limit the over-proliferation of the symbiotic bacteria and maintain gut homeostasis. Normally, when mosquitoes ingest sugar, the symbiotic bacteria in the midgut are kept at a relatively low level (Pumpuni et al., 1996; Wang et al., 2012). This healthy status requires mosquitoes to deploy basal immunity to maintain a symbiotic relationship with the bacteria (Wei et al., 2017). The gut microbiota and the host mosquito have adapted to coexist during long-term adaptive coevolution, which may be the reason why the gene expression patterns of the *Serratia* Y1 and J1 mosquitoes are similar when they feed on sugar.

After ingestion of a *Plasmodium* infected blood meal by the mosquito, the parasite undergoes sexual development in the midgut lumen to form a motile ookinete that invades the midgut epithelium around 24–26 h after blood feeding (Sinden and Billingsley, 2001; Baton and Ranford-Cartwright, 2005). The bacterial numbers in the mosquito midgut also increase by 100- to 1,000-fold 24 h after a blood meal (Cirimotich et al., 2011), activating the mosquito immune system to limit the over-proliferation of gut bacteria (Dong et al., 2009). We found that the c-type lectins (CTLs) and CLIP serine proteases were were up-regulated in the *Serratia* Y1 group. Many CLIP family genes have been showed to affect *Plasmodium* development. CLIPA2, A5 and A7 suppress parasite melanization, and CLIPA2 and CLIPA5 interact synergistically to block ookinete invasion (Barillas-Mury, 2007), while CLIPB3, B4, B8 and B17 promote ookinete invasion (Volz et al., 2006). CLIPB14 and CLIPB15 are also involved in killing *Plasmodium* ookinetes and participate in the response against bacteria (Volz et al., 2005). Interestingly, expression of CTL4 was severely down-regulated in the *Serratia* Y1 challenged mosquitoes at 24 h post-blood meal. CTL 4 and CTLMA 2 are present in the hemolymph and up-regulated 24 h after blood ingestion and can protect the rodent *Plasmodium* ookinetes from destruction (Osta et al., 2004). FBN9, a member of the fibrinogen domain immunolectin family (FBN), was up-regulated in the *Serratia* Y1 fed mosquitoes at 24 h post-blood meal. A previous study suggested that FBN9 interacts with gram-positive and gram-negative bacteria and also affects *Plasmodium* development (Dong and Dimopoulos, 2009). The complement-like protein TEP1 and the leucine-rich repeat (LRR) protein APL1, which mediate lysis of *Plasmodium* parasites in the mosquito midgut (Blandin et al., 2008; Garver et al., 2009; Dong et al., 2011), were also up-regulated in the *Serratia* Y1 group at 24 h post-blood meal. Other factors including LRRD7, TEP12, NOX5, PGRP-LB, NOS and PGRP-LC, which had been shown to affect *Plasmodium* infection (Luckhart et al., 1998; Dong et al., 2006; Meister et al., 2009; Oliveira Gde et al., 2012), were also up-regulated in the *Serratia* Y1 group at 24 h post-blood meal. We further demonstrated that silencing of *TEP1*, *FBN9*, or *LRRD7* markedly reverses the parasite inhibition conferred by *Serratia* Y1 infection. Previous studies reported that these three genes can strongly influence both *P. falciparum* and *P. berghei* development (Blandin et al., 2004; Dong et al., 2006). Taken together, *Serratia* Y1 elicited a strong immune response in the mosquito midgut after taking a blood meal and knock-down of several highly elicited immune genes induced a significant increase of *P. berghei* oocysts, further confirming that *Serratia* Y1 interferes with *P. berghei* development by eliciting the mosquito’s immune response.

In summary, our data shows that *Serratia* Y1 inhibits *Plasmodium* development through stimulation of the mosquito immunity. Our work establishes an important framework of knowledge for further investigations into the molecular interactions between gut bacteria, *Anopheles* mosquitoes and pathogens, which would enhance our understanding of how gut bacteria inhibit *Plasmodium* development in the midgut of mosquitoes.

## Data Availability

RNA sequencing datasets from this work were deposited in the National Center for Biotechnology Information Sequence Read Archive (accession no. PRJNA520745).

## Author Contributions

SW conceived the study. SW and LB designed the experiments. LB performed the majority of experiments and generated fluorescent strains and performed transcriptome analysis. LB and GW performed RNAi analysis. LW and JV-R discussed results and provided advice. LB and SW analyzed the data. JV-R edited the manuscript. LB, LW, and SW wrote the manuscript.

### Conflict of Interest Statement

The authors declare that the research was conducted in the absence of any commercial or financial relationships that could be construed as a potential conflict of interest.

## Footnotes

^1^https://www.vectorbase.org/organisms/anopheles-stephensi

^2^http://cole-trap-nell-lab.github.io/cufflinks/

^3^ftp://ftpmips.gsf.de/FGDB/

^4^http://www.cbs.dtu.dk/services/SignalP/
