# Supplementary material for: A Gut Symbiotic Bacterium Serratia marcescens Renders Mosquito Resistance to Plasmodium Infection Through Activation of Mosquito Immune Responses
Source: Front Microbiol. 2019 Jul 18;10:1580. doi: 10.3389/fmicb.2019.01580 (PMC6657657; doi:10.3389/fmicb.2019.01580)
Supplement: Supplementary file 1 [file Data_Sheet_1.PDF]

Supplementary Material

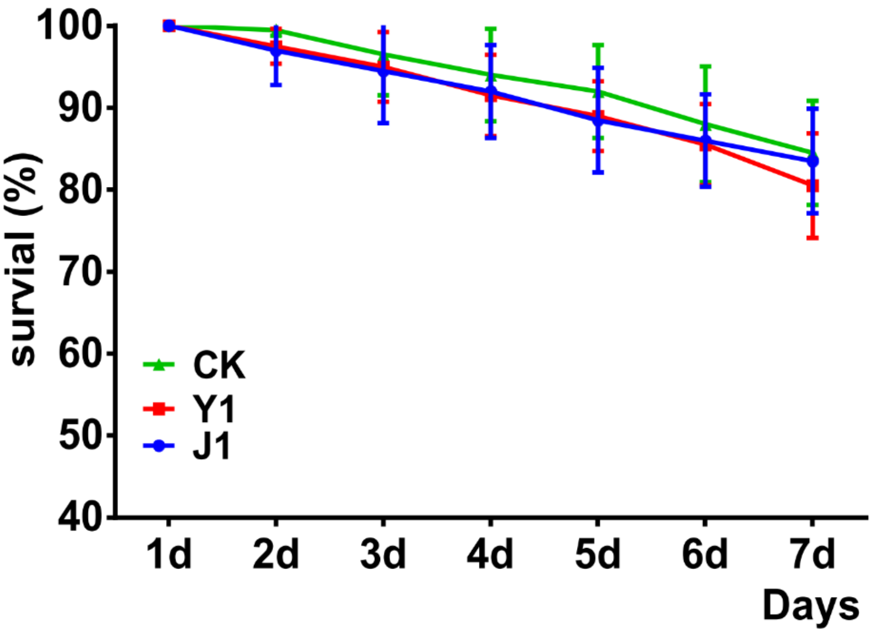

Supplementary Figure 1. Effect of *Serratia* Y1 and J1 on mosquito survival. Two day-old adult *An. stephensi* mosquitoes were allowed to feed for 24 h on a cotton pad moistened with 5%sucrose solution containing 10^7 bacteria/ml or 5% sugar alone (CK). The bacteria-fed female mosquitoes were then starved for 8 h and allowed to feed on a bloodmeal. Mosquitoes were maintained at 27 °C with 10% sucrose solution. Mosquito survival was monitored daily. There was no significant difference in survivorship between *Serratia* Y1- or J1-fed mosquitoes and sugar alone-fed controls.

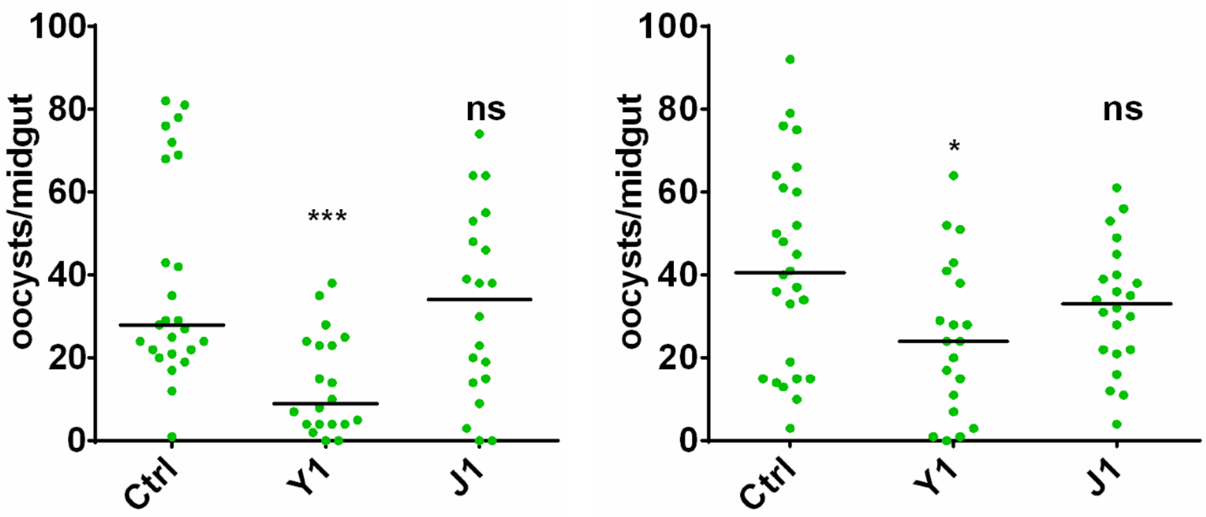

Supplementary Figure 2. Oocyst infection intensity in *An. stephensi* mosquitoes colonized with *Serratia* Y1 or J1 after feeding on *P. berghei* mCherry infected mice. The results represent two independent experiments and confirm the phenotypes shown in Fig. 1B. Each dot represents the oocysts number from individual midguts, and the horizontal lines indicate the median number of oocysts. The Mann-Whitney test was used to determine significance in oocysts numbers, * $P < 0.05$, *** $P < 0.001$.

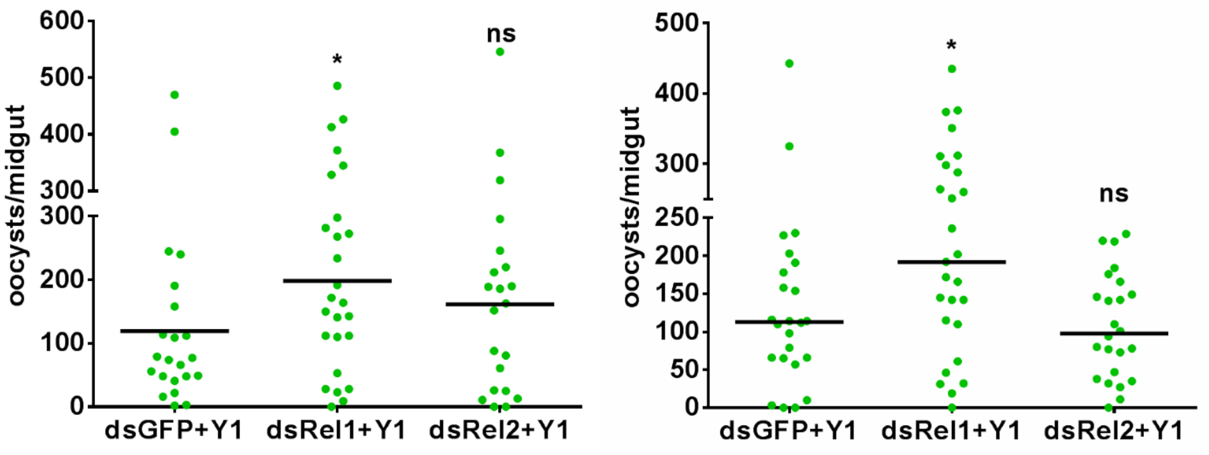

Supplementary Figure 3. Effect of *Rel1* and *Rel2* silencing on the *Serratia* Y1-mediated anti-*Plasmodium* activity. The results represent two independent experiments and confirm the phenotypes shown in Fig. 2. *Rel1* and *Rel2* were silenced in *An. stephensi* mosquitoes by systemic injection of dsGFP, dsRel1 or dsRel2 RNA. The injected mosquitoes were fed on a sugar meal containing *Serratia* Y1. Three days later, all mosquito groups were allowed to feed on the same *P. berghei* infected mouse. The injected double-stranded RNA (ds) and presence (Y1) of *Serratia* Y1 are indicated below each column. Each dot represents the number of oocysts from an individual midgut, and the horizontal lines indicate the median number of oocysts. The Mann-Whitney test was used to determine significance in oocysts numbers, *$P < 0.05$, ns: no significant.

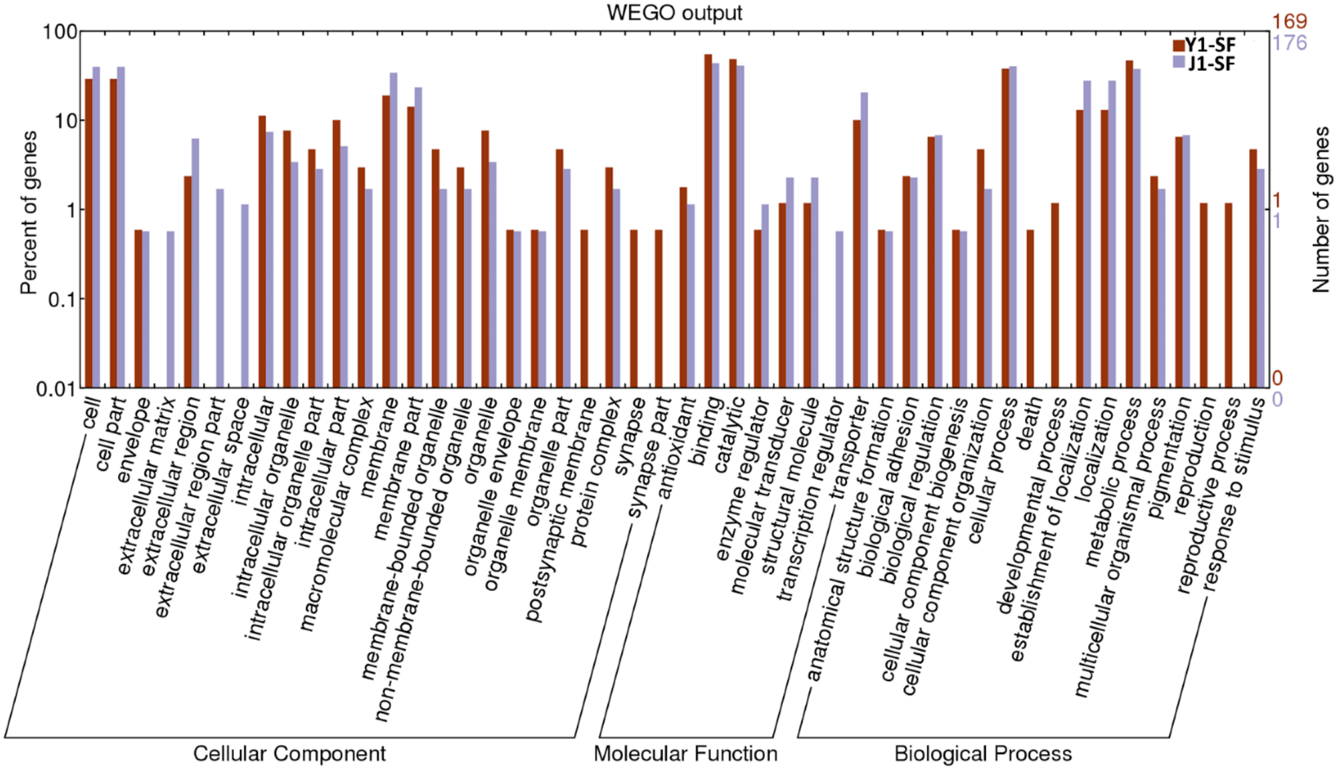

Supplementary Figure 4. GO term enrichment analysis of cellular component, molecular function and biological process of genes differentially regulated in the midgut of mosquitoes challenged with *Serratia* Y1 or J1 during sugar feeding.

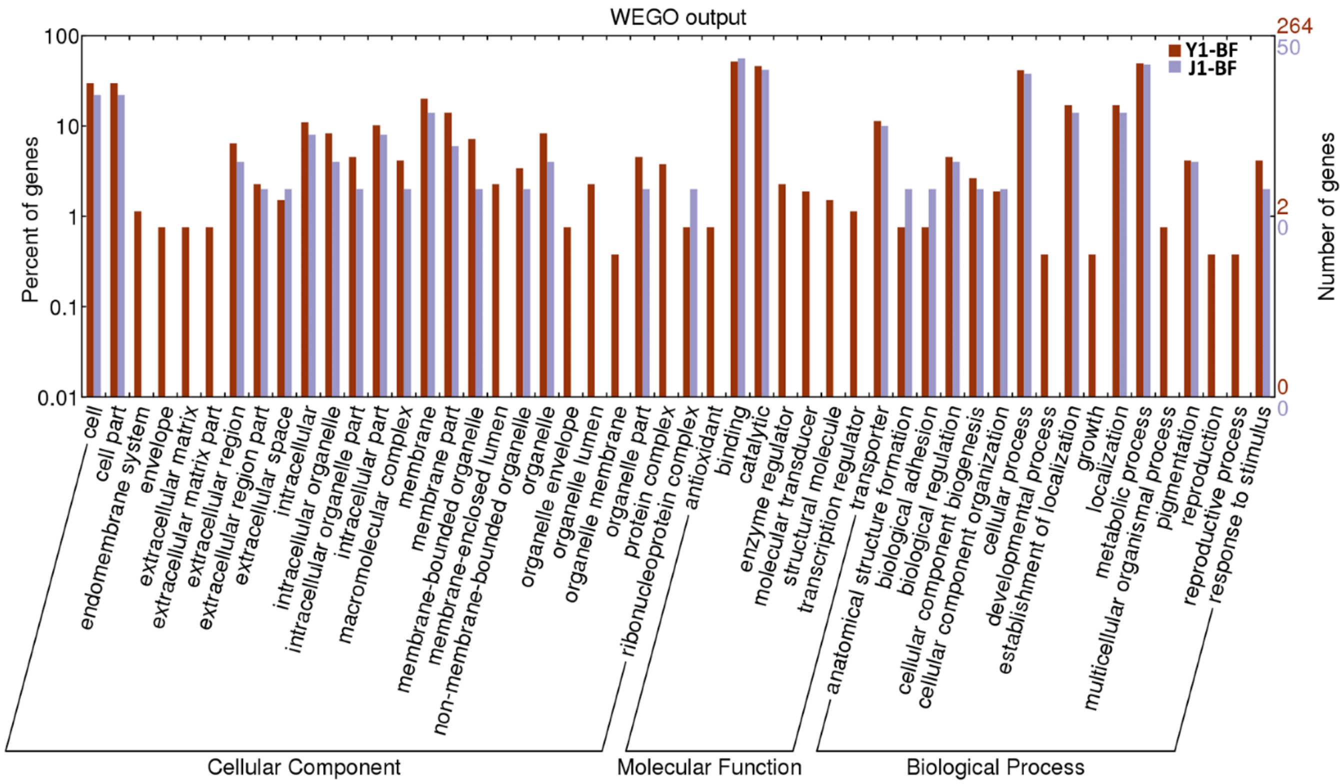

Supplementary Figure 5. GO term enrichment analysis of cellular component, molecular function and biological process of genes differentially regulated in mosquitoes challenged with *Serratia* Y1 and J1 at 24 h post blood meal.

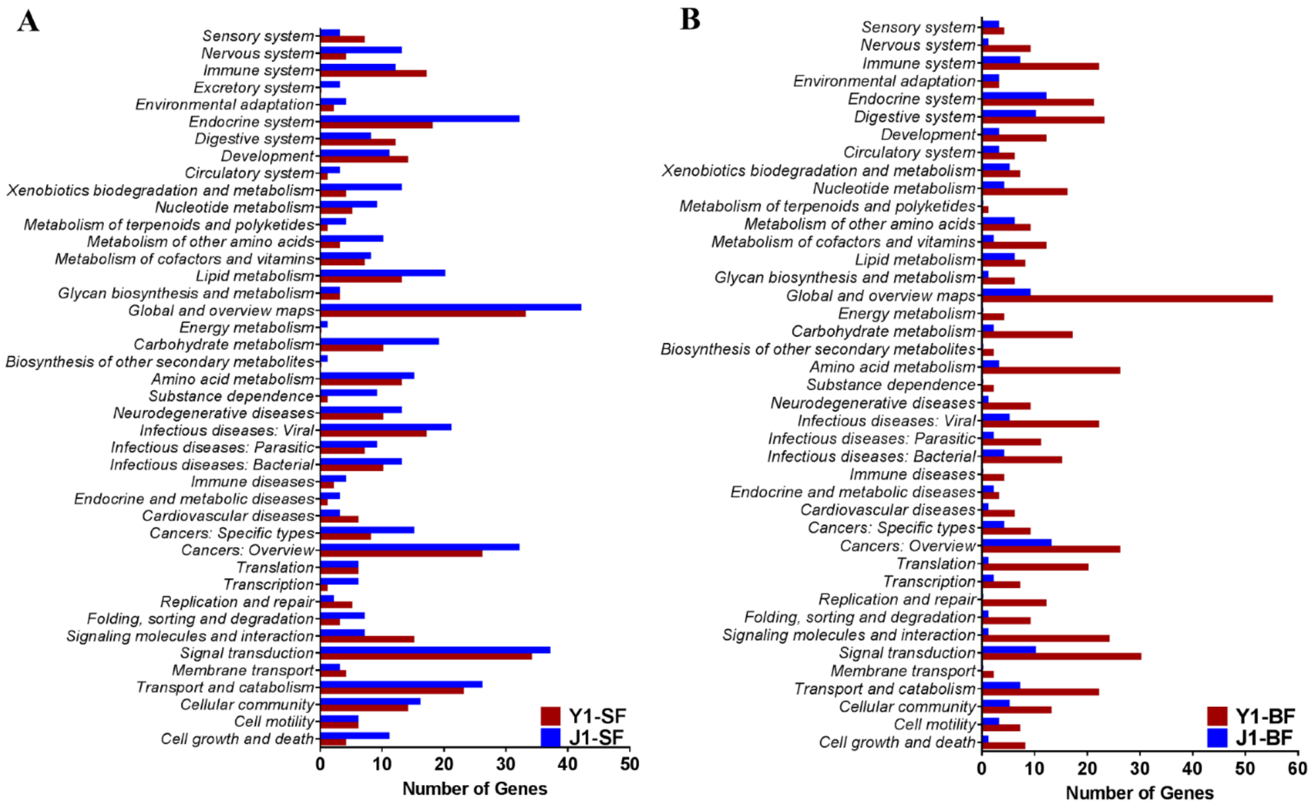

Supplementary Figure 6. KEGG annotation analysis of genes differentially regulated in the midgut of mosquitoes challenged with *Serratia* Y1 and J1 before and 24 h post blood meal.

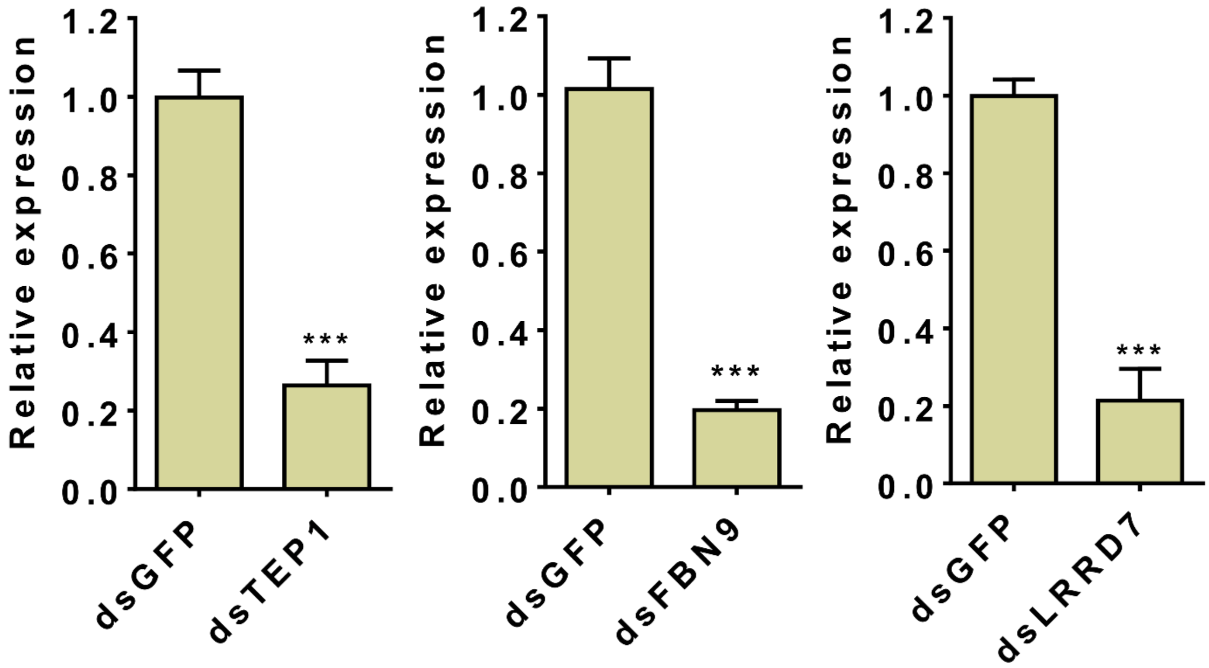

Supplementary Figure 7. Analysis of gene silencing efficiency of *TEP1*, *FBN9* and *LRRD7* in adult female *A. stephensi* mosquitoes. Systemic injection of *TEP1* dsRNA (dsTEP1), dsFBN9 and dsLRRD7 significantly reduced their transcript levels. Error bars represent the mean ± SEM. Pooled data from three independent experiments. *** $P < 0.001$ (Student's *t* test).

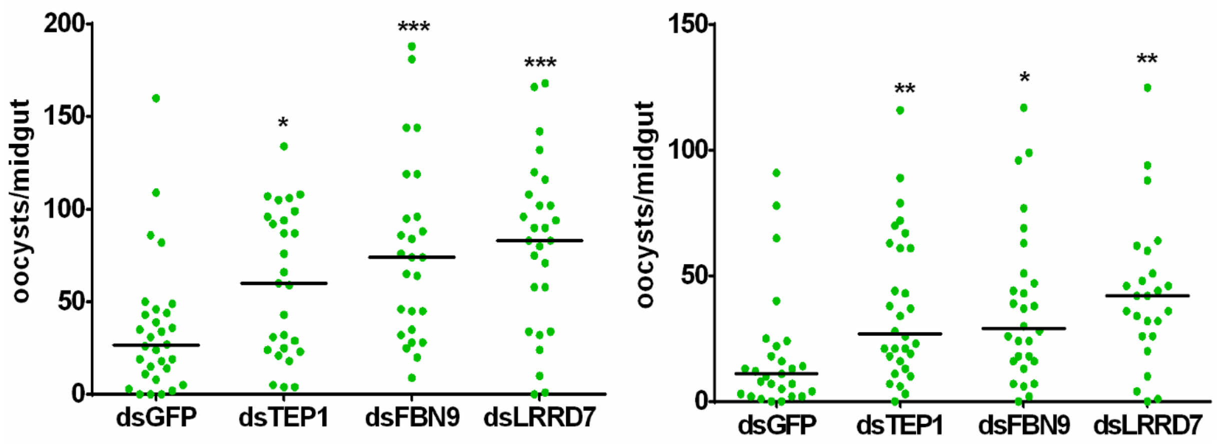

Supplementary Figure 8. Oocyst loads following silencing of *TEP1*, *FBN9* and *LRRD7* genes. The results represent two independent experiments and confirm the phenotypes shown in Fig. 6. *An. stephensi* female mosquitoes were injected with dsRNA solution for *TEP1*, *FBN9*, *LRRD7* or *GFP* as a control. Injected mosquitoes were colonized with *Serratia* Y1 and then fed on a *P. berghei* infected mouse 3 days after dsRNA injection. Each dot represents the number of oocysts from individual midguts, and the horizontal lines indicate the median number of oocysts. The Mann-Whitney test was used to determine significance in oocysts numbers, * $P < 0.05$, ** $P < 0.01$, *** $P < 0.001$.

Supplementary Table 1. Summary of RNA-seq data from six RNA libraries

Libraries	Total Clean Reads	Total Mapping Ratio	Uniquely Mapping Ratio
Y1_0h	44,081,078	70.10%	68.30%
Y1_24h	44,756,220	62.64%	59.64%
J1_0h	42,503,790	46.79%	43.35%
J1_24h	41,546,786	71.98%	69.09%
Ctrl_0h	41,501,222	56.05%	53.41%
Ctrl_24h	38,272,772	48.32%	44.49%

Supplementary Table 2. Primer sequences used for qPCR analysis

Primers	Sequences
**APL1A-F**	GATTAGCGGCGACAGCG
**APL1A-R**	TCGGTCAAGGTAGGGGAAGT
**DEF1-F**	AGTCGTGGTCCTGGCGGCTCT
**DEF1-R**	ACGAGCGATGCAATGCGCGGCA
**CEC1-F**	GGAAGCGGGACGCCTGAA
**CEC1-R**	CCTTGACACCTGCCACCACC
**S7-F**	TCGGTTCCAAGGTGATCAAAGC
**S7 -R**	AGCGCGGTCTCTTCTGCTTGT
**GAM1-F**	GTACGTCAGCCGGAAGGGAG
**GAM1-R**	CGTAATGAACGAGGACGAACAGC
**LRRD7-F**	ACAGTACGGCGTTCAAGCG
**LRRD7-R**	AAGGGCACCCACGAAACA
**TEP1-F**	TCAGATGCGCTATCGCCAGT
**TEP1-R**	GCTCAGATAGGCCATTGCATT
**ATT-F**	AAAGCCAGAGCGGCAACAC
**ATT-R**	TCAGTAACCGTGCGTGAAAGTC
**FBN9-F**	AACAATCTGACCGCACTGC
**FBN9-R**	TGTGACGCATTCCCTGTAG

Supplementary Table 3. Primer sequences used for dsRNA production

Primers	Sequences
dsGFP-F	TAATACGACTCACTATAGGGGTGAGCAAGGGCGAGGAGCTGT
dsGFP-R	TAATACGACTCACTATAGGGTTACTTGTACAGCTCGTCCAGCCG
dsRel1-F	TAATACGACTCACTATAGATCAACAGCACCACCATGAGC
dsRel1-R	TAATACGACTCACTATAGTCGAAGAATCGCACCTTAATATCTTCC
dsRel2-F	TAATACGACTCACTATAGCGGAGAAGTCGAAGAAAACGTTC
dsRel2-R	TAATACGACTCACTATAGCATAGGCACACCTGGTTAAGGT
dsLRRD7-F	TAATACGACTCACTATAGGGACCGACACTGTTCCAAACG
dsLRRD7-R	TAATACGACTCACTATAGGGATGGCATTGCTTTCCACC
dsTEP1-F	TAATACGACTCACTATAGGGCGCTAATCAACAAGGCGAC
dsTEP1-R	TAATACGACTCACTATAGGGATCACGAACGACAGCAAGG
dsFBN9-F	TAATACGACTCACTATAGGGTCTACAGGGAATGCGTCAC
dsFBN9-R	TAATACGACTCACTATAGGGTCTACAGGGAATGCGTCAC
